# Combined intravoxel incoherent motion diffusion-weighted MR imaging and magnetic resonance spectroscopy in differentiation between osteoporotic and metastatic vertebral compression fractures

**DOI:** 10.1186/s13018-019-1350-3

**Published:** 2019-09-05

**Authors:** Hui Tan, Hui Xu, Feifei Luo, Zhaoguo Zhang, Zhen Yang, Nan Yu, Yong Yu, Shaoyu Wang, Qiuju Fan, Yue Li

**Affiliations:** 1 Institute of Medical Technology, Shaanxi University of Chinese Medicine, Xianyang, China; 2 Department of Radiology, Affiliated Hospital of Shaanxi University of Chinese Medicine, Xianyang, China; 3 Department of Medical Imaging, The First Affiliated Hospital of Xi’an Jiaotong University, Xi’an, China; 4 Faculty of Dentistry, University of Toronto, Toronto, Ontario Canada; 5 Centre for the Study of Pain, University of Toronto, Toronto, Ontario Canada; 6 The Key Laboratory of Biomedical Information Engineering of Ministry of Education, Institute of Biomedical Engineering, School of Life Science and Technology, Xi’an Jiaotong University, Xi’an, China; 7 Siemens Healthineers, Shanghai, China

**Keywords:** Vertebra, Fractures, Osteoporotic, IVIM-DWI, MRS

## Abstract

**Purpose:**

Our purpose was to combine intravoxel incoherent motion diffusion-weighted MR imaging (IVIM-DWI) and magnetic resonance spectroscopy (MRS) to differentiate osteoporotic fractures from osteolytic metastatic vertebral compression fractures (VCFs).

**Methods:**

A total of 70 patients with VCFs were included and divided into two groups, according to their causes of fractures based on pathological findings or clinical follow-up. All patients underwent conventional sagittal T1WI, T2WI, STIR, IVIM-DWI, and single-voxel MRS. The diffusion coefficient (D), pseudo diffusion (D*), and perfusion fraction (f) parameters from IVIM-DWI and the lipid water ratio (LWR) and fat fraction (FF) parameters from MRS were obtained and compared among groups. Furthermore, the diagnostic performance of MRS, IVIM-DWI, and IVIM-DWI combined with MRS for differentiation between osteoporotic and osteolytic metastatic VCFs was assessed by using receiver operating characteristic (ROC) curve analysis.

**Results:**

Compared with the osteoporotic group, the metastatic group had significantly lower values for f, D, and FF, but higher D* (all *P* < 0.05). The area under the receiver operating characteristic (ROC) curve of MRS, IVIM-DWI, and IVIM-DWI combined with MRS were 0.73, 0.88, and 0.94, respectively. Among these, the IVIM-DWI combined with MRS showed the highest sensitivity, specificity, and accuracy, which are 90.63% (29/32), 97.37 % (37/38), and 94.29% (66/70), respectively.

**Conclusions:**

IVIM-DWI combined with MRS can be more accurate and efficient for differentiation between osteoporotic and osteolytic metastatic VCFs than single MRS or IVIM-DWI.

## Introduction

Vertebral compression fractures (VCFs) are common and occurred frequently among the elderly, which are caused by osteoporosis. However, the spine is also a frequent location of metastatic that may result in pathologic fractures in one third of patients with cancer. Regular diagnosis is very important for the option of treatments and prognosis for patients; moreover, the unnecessary vertebral biopsy can be evitable in patients with osteoporotic VCFs [1]. Though useful, morphological features only based on CT may be misleading. Magnetic resonance imaging (MRI) plays an increasingly vital role in differentiating osteoporotic from metastatic VCFs. A T1- or T2-weighted imaging and short-T1 inversion recovery (STIR) sequence are performed for the clinical assessment with low specificity [2].

Diffusion-weighted imaging (DWI) has proven to be useful in oncological imaging for tumor detection and characterization [3–5]. However, the results were confusing by the variability among ADC measurements. Recently, the intravoxel incoherent motion (IVIM) DWI has been used to quantitatively assess the microscopic translational motion [6]. Furthermore, the application of IVIM-DWI-based quantitative analysis for evaluating cancers is increasing. The distinct advantage of IVIM-DWI is obtaining the sufficient parameters about diffusion and perfusion simultaneously [7]. The perfusion-related parameters of IVIM-DWI have been performed among the brain, prostate gland, liver, and breast [8–11]. To our knowledge, few studies were performed on vertebrae by using IVIM-DWI [12–14]. Regular bone marrow in the axial skeleton has fat and water components (red marrow has about 40% fat component, while yellow marrow has 80% fat component). In tumor infiltration, the lipid is replaced by tumor cells [15]. Previous studies have shown that 1H-MRS was able to detect fat component from the bone marrow. However, few studies have been reported for fat content quantification of fat component from the vertebral bone marrow in patients with osteoporosis or metastasis using 3T MRI [16, 17].

In this study, our purpose was to examine whether IVIM-DWI combined with MRS as a noninvasive method can be used to differentiate osteoporotic fractures from metastatic VCFs.

## Methods and materials

### Participants

**Fig. 1:**
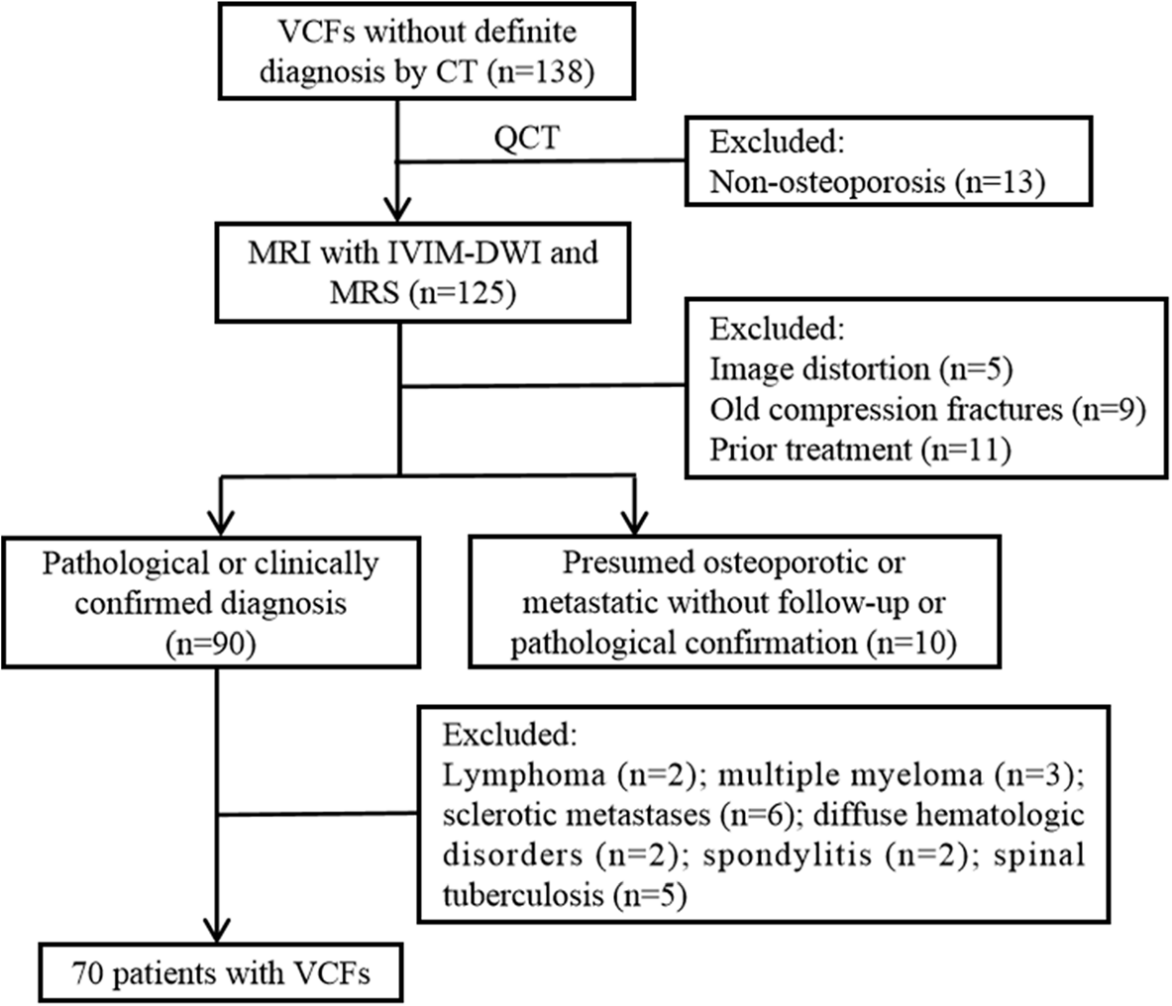
A flowchart showing the selection of this study population. VCFs, vertebral compression fractures; QCT, quantitative computed tomography; IVIM-DWI, intravoxel incoherent motion diffusion-weighted MR imaging; MRS, magnetic resonance spectroscopy

All consecutive patients (*n* = 138) in the orthopedics suffered from VCFs and underwent CT examination without definite diagnoses and were recruited in this study between August 2016 and July 2017. Then, all participants underwent quantitative CT bone densitometry examination and received 3.0-T spinal MRI, including conventional sagittal T1WI, T2WI, STIR, and IVIM diffusion-weighted imaging and ^1^H-MRS. Inclusion criteria for all patients were (i) an acute or subacute vertebral fractures for less than 3 months, (ii) bone marrow edema at the fracture sites, (iii) aged 18 years or older, (iv) no MRI contraindications (e.g., cardiac pacemaker, aneurysm clip, claustrophobia), and (v) osteoporosis (with a T score below 2.5). Among the 138 patients, the following exclusion criteria were applied: (i) Unsatisfactory image quality or an artifact caused by a metal device (*n* = 5), (ii) old compression fractures (*n* = 9), (iii) chemotherapy or radiation therapy before MRI examination (*n* = 11), (iv) patients with presumed osteoporotic or metastatic without more than 3 months follow-up or did not have pathological confirmation (*n* = 10), and (v) primary vertebral tumors (e.g., lymphoma(*n* = 2) and multiple myeloma (*n* = 3)), sclerotic metastases (*n* = 6), diffuse hematologic disorders (*n* = 2), spondylitis (*n* = 2), spinal tuberculosis (*n* = 5). Therefore, a total of 70 patients with VCFs were included, and the onset time for the fractures patients suffered from was ranged from 6 h to 15 days before MR imaging (Fig. 1). All the participants gave written informed consent in person approved by a local institutional review board and conducted in accordance with the Declaration of Helsinki.

All patients were divided into osteoporotic group and metastatic group according to their causes of the vertebral fracture, which were determined by the gold standard histology biopsy, combined with more than 3 months follow-up MRI or CT. The morphological signs of malignant compression fractures include vertebral compression and flattening, and the posterior marginal cortical of the vertebral body is bulge, pedicle invasion, spinal epidural, and paravertebral soft tissue mass formation [18].

The osteoporotic group consisted of 38 patients with osteoporotic VCFs, including 2 cervical vertebrae, 20 thoracic vertebrae, and 16 lumbar vertebrae. The diagnosis criteria and distributions for the osteoporotic fracture were (1) surgery and histopathologic examination (*n* = 2), (2) no primary tumor, (3) follow-up CT (*n* = 17), and/or MRI (*n* = 19) for more than 3 months: edema disappeared, non-morphologic signs of malignancy (e.g., bone destruction, soft tissue swelling), as well as the disappearance of clinical pain.

The metastatic group consisted of 32 patients with malignant VCFs, including cervical vertebra (*n* = 6), thoracic vertebra (*n* = 16), and lumbar vertebra (*n* = 10). The sources of the primary neoplasms were lung cancer (*n* = 10), breast cancer (*n* = 7), prostate cancer (*n* = 6), renal cell carcinoma (*n* = 3), colorectal cancer (*n* = 2), ovarian cancer (*n* = 2), pancreatic cancer (*n* = 1), and thyroid carcinoma (*n* = 1). The diagnosis criteria and distributions for the metastatic fracture were (1) primary malignant tumor in other sites (*n* = 32), (2) CT-guided biopsy (*n* = 3), (3) follow-up CT (*n* = 12), and/or MRI (*n* = 17) for more than 3 months: the persistent of bone marrow edema, morphologic signs of malignancy (e.g., bone destruction, soft tissue swelling), and the persistent of clinical pain.

### MRI acquisition protocols

The MRI scans were acquired with the use of 3.0 T MRI scanner (MAGNETOM Skyra, Siemens AG, Erlangen, Germany) with a quadrature spine surface coil. The routine MRI sequences were obtained firstly including T1-weighted (echo time (TE) = 10 ms, repetition time (TR) = 550 ms) and T2-weighted (TE = 110 ms, TR = 4000 ms). The STIR (TE = 65 ms, TR = 3500 ms, inversion time = 180 ms) images of 18 sagittal slices with a slice thickness of 4 mm were acquired (field of view (FOV) = 256 mm × 256 mm, matrix size = 256 × 256).

The IVIM-DWI sequence was performed with the following parameters: TR = 1600 ms, TE = 72 ms, slice thickness = 5 mm, FOV = 340 mm × 340 mm, matrix size = 256 × 256, and *b* values = 0, 50, 100, 150, 200, 400, 600, and 800 s/mm^2^, respectively. The mean acquisition time of the IVIM sequence was 6:54 min.

Similarly, MRS was performed using the point resolved spectroscopy (PRESS) sequence with the following parameters: TR = 2000 ms, TE = 30 ms, NEX = 64, bandwidth = 2000 Hz, flip angle = 90°, without water suppression, and total scan time of 4:27 min. The dimensions of voxels were varied according to the configuration of the fracture. The MRS voxels were positioned centrally within the STIR hyperintensity of the fractured vertebrae, avoiding the cystic degeneration and necrosis. On average, the voxels were comparable at 3.0 ± 0.5 cm^3^.

### Preprocessing

All MR images were retrospectively identified by two independent radiologists (with 3 and 9 years of experience in musculoskeletal imaging, respectively), who were blinded to the purpose of this study. For each patient, the acquired MRS images were pre-processed by applying the Siemens Syngo.via workstation. The LC-Model program was designed to determine the relative peak areas of the signals of water at 4.7 ppm and lipid at 1.3 ppm. Both parameters of lipid water ratio (LWR) and fat fractions (FF) were calculated according to the following equations:

$$ {\displaystyle \begin{array}{l}\mathrm{LWR}={\mathrm{S}}_{\mathrm{Lipid}}/{\mathrm{S}}_{\mathrm{Water}}\\ {}\mathrm{FF}=\mathrm{LWR}/\left(\mathrm{LWR}+1\right)\end{array}} $$

where *S*_lipid_ is the area under the lipid peak, and *S*_water_ is the area under the water peak.

The IVIM-derived parameters for each patient were estimated with MITK Diffusion (release 2015.05, www.mitk.org, an open-source software), which yielded values for *D*, D* and *f*. Regions of interest (ROIs, 75.40~176.60 mm^2^) were manually drawn independently by the two radiologists on each lesion which exhibited the maximal lesion area on IVIM parameter maps corresponding to the MRS voxel directly.

### Statistical analysis

Firstly, inter-observer agreements were evaluated by calculating intraclass correlation coefficients (ICCs). The value *r* was estimated as follows: 1 denotes perfect agreement, 0.81–0.99 denotes almost perfect agreement, 0.61–0.80 denotes substantial agreement, 0.41–0.60 denotes moderate agreement, 0.21–0.40 denotes fair agreement, and ≤ 0.20 denotes slight agreement. Secondly, the difference of parameters between groups was performed using two-sample *t* tests. Significance was set at *P* < 0.05 corrected for multiple comparisons. Finally, multivariate logistic regression was adopted to identify independent factors for differential diagnosis of osteoporotic and osteolytic metastatic fractures by the MRS, IVIM-DWI, and IVIM-DWI combined with MRS. The validity of parameters in the diagnosis of metastasis was evaluated by applying receiver operating characteristic (ROC) analysis, which calculated the value of the sensitivity, specificity, and diagnostic accuracy. Furthermore, the areas under the ROC curve (AUC) were compared for significant difference between the MRS, IVIM-DWI, and IVIM-DWI combined with MRS.

## Results

### Patients’ characteristics

The osteoporotic group consisted of 38 patients with osteoporotic VCFs (male 16, mean age 65.2), and there were 32 patients with malignant VCFs in the metastatic group (male 18, mean age 63.2). There were no differences in age (*P* > 0.05) and gender (*P* > 0.05) between two groups. All demographic characteristics for patients were presented in Table 1.

**Table 1:** Summary of demographics characteristics of all patients

	Osteoporotic group	Metastatic group	*P* value
Patients (*n*)	38	32	
Gender (male/female)	16/22	18/14	0.174
Age (years)	65.2 ± 9.0	63.2 ± 13.6	0.501

### ICC of imaging parameters

Inter-observer agreement of imaging parameters *D* and D* were all perfect agreement in osteoporotic and metastatic groups (all ICC> 0.81, *P*> 0.05, Table 2).

**Table 2:** ICCs of imaging parameters

Parameters		Reader 1	Reader 2	ICC (95% CI)	*P* value
*D* (× 10^−3^ mm^2^/s)	Osteoporotic	1.65 ± 0.34	1.69 ± 0.36	0.879 (0.768, 0.937)	0.642
	Metastatic	1.09 ± 0.98	0.98 ± 0.75	0.968 (0.934, 0.984)	0.361
D* (× 10^−3^ mm^2^/s)	Osteoporotic	24.52 ± 8.28	27.35 ± 10.05	0.869 (0.710, 0.936)	0.486
	Metastatic	51.61 ± 14.37	56.08 ± 12.63	0.876 (0.746, 0.940)	0.554
*f*	Osteoporotic	0.17 ± 0.09	0.16 ± 0.08	0.957 (0.917, 0.978)	0.513
	Metastatic	0.10 ± 0.05	0.12 ± 0.05	0.856 (0.705, 0.930)	0.678

Data in parentheses are 95% confidence intervals

*ICC* intraclass correlation coefficient, *D* pure diffusion coefficient. D* pseudo diffusion coefficient, *f* perfusion fraction

### Parameters’ differences between groups

**Fig. 2:**
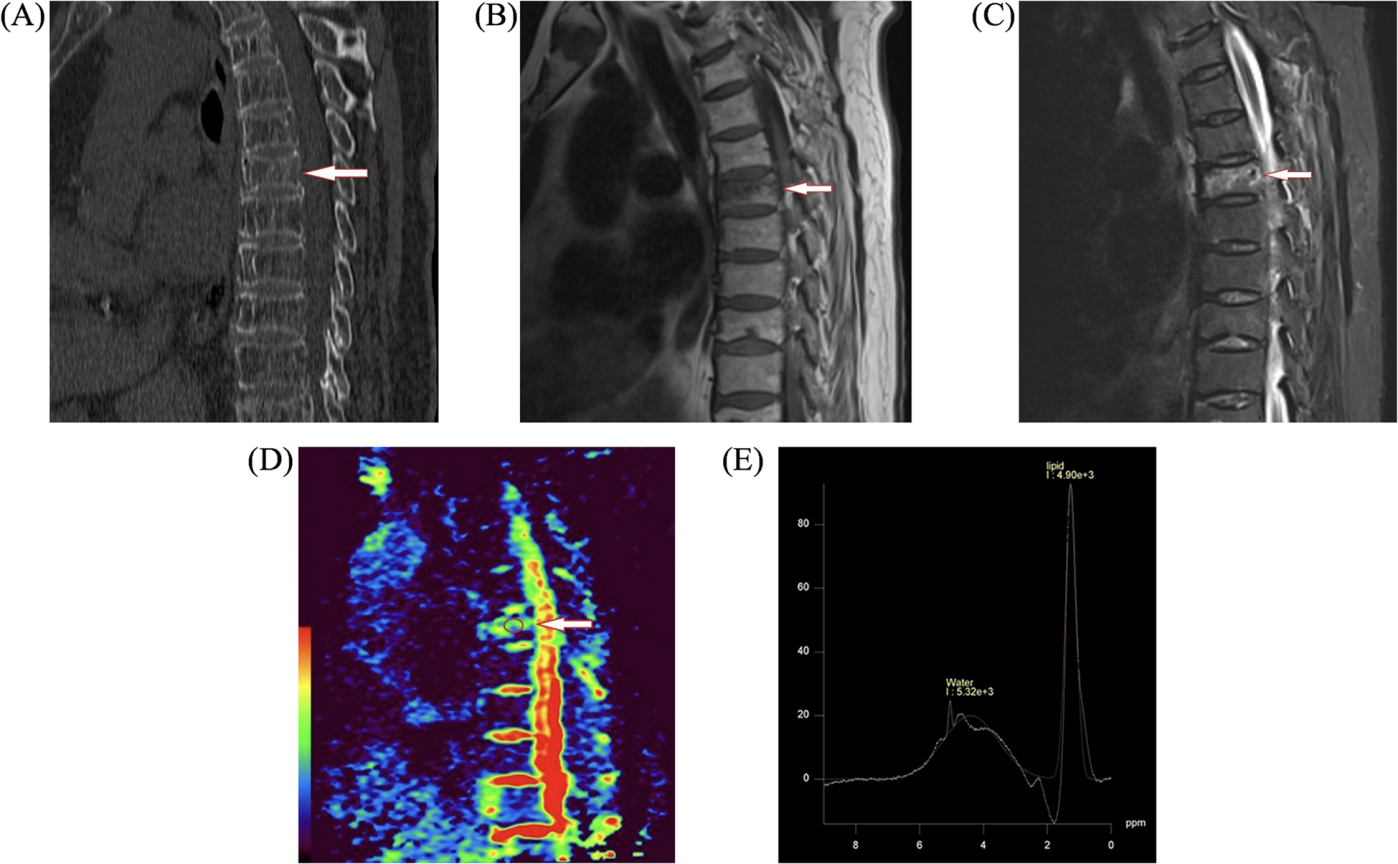
A 67-year-old woman with osteoporotic fracture of T7 vertebra. **a** Mid sagittal CT shows vertebral compression changes and osteoporotic changes (arrow). **b**, **c** T1-weighted and T2-weighted images. The lesion is hypointense on T1-weighted image (arrow) and hyperintense on T2-weighted image (arrow). **d** IVIM-DWI image, high signal, and regions of interest were placed within lesion (circle), *f* = 0.128, *D* = 1.65 × 10^−3^ mm^2^/s, D* = 1.84 × 10^−2^ mm^2^/s. **e** MRS, LPA = 4900, WPA = 5320, lipid fraction of 47.79 at T7

**Fig. 3:**
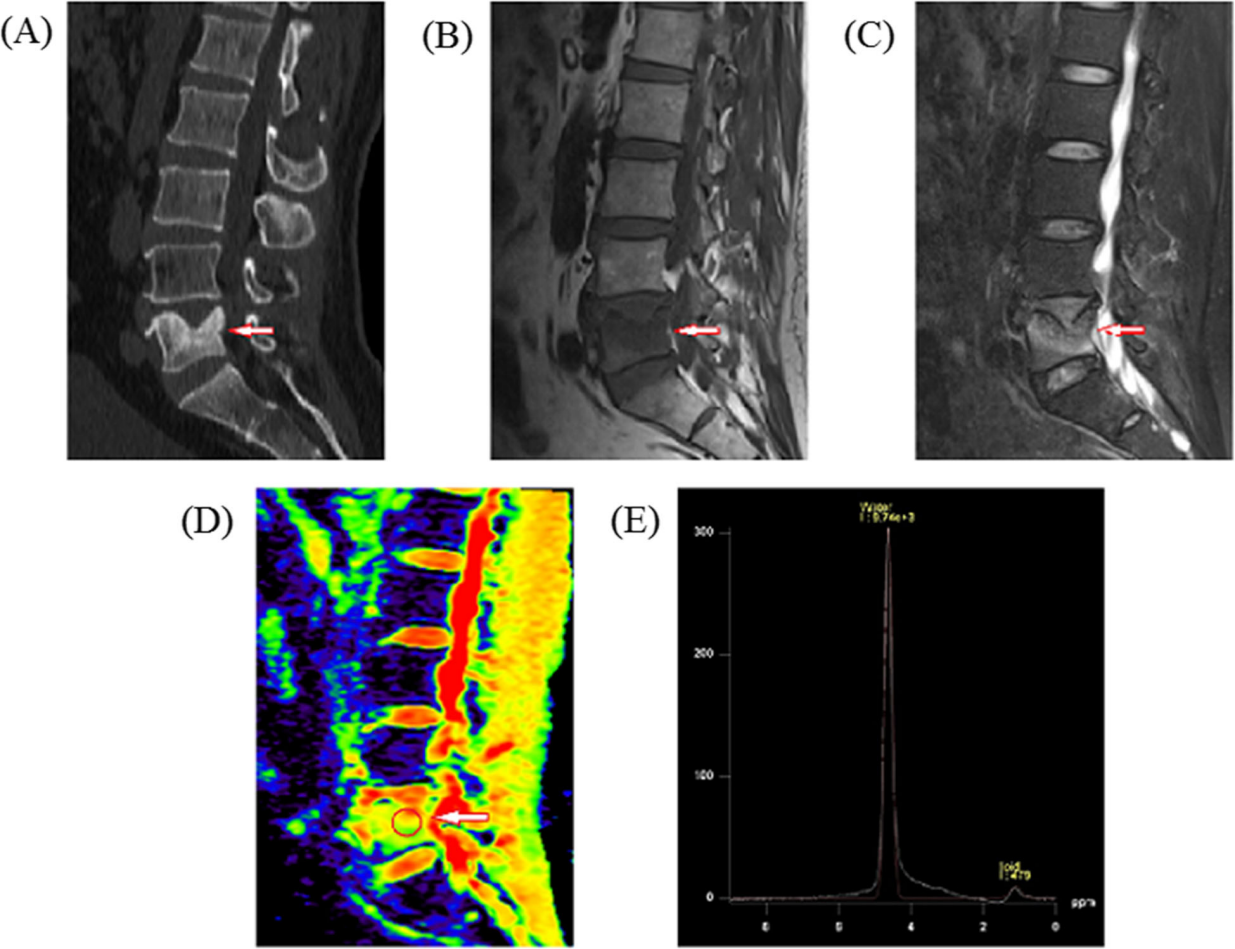
A 59-year-old man with lung neoplasm of L5 vertebra. **a** Mid sagittal CT showed vertebra bone destruction (arrow). **b**, **c** T1-weighted and T2-weighted images. The lesion is hypointense on T1-weighted image (arrow) and hyperintense on T2-weighted image (arrow). **d** IVIM-DWI image, high signal, and regions of interest were placed within lesion (circle), *f* = 0.092, *D* = 1.17 × 10^−3^ mm^2^/s, D* = 6.44 × 10^−2^ mm^2^/s; e MRS, LPA = 479, WPA = 9740, lipid fraction of 4.69 at L5 metastatic fracture

Compared with the osteoporotic group, the *f*, *D*, and FF values in the metastatic group were significantly lower, whereas the D^*^ value was significantly higher (all *P* < 0.05, Table 3). The examples of an osteoporotic fracture and a metastatic fracture were displayed in Fig. 2 and Fig. 3, respectively.

**Table 3:** Parameters differences between groups

Parameters	Osteoporotic group (*n* = 38)	Metastatic group (*n* = 32)	*t* value	*P* value
LWR (%)	42.22 ± 21.20	27.73 ± 18.45	3.02	0.004
FF (%)	24.73 ± 7.68	14.95 ± 7.26	5.44	0.000
*D* (× 10^−3^ mm^2^/s)	1.67 ± 0.35	1.04 ± 0.95	3.79	0.000
D* (× 10^−3^ mm^2^/s)	25.81 ± 10.02	53.84 ± 15.61	− 8.75	0.000
f	0.16 ± 0.08	0.11 ± 0.06	3.28	0.002

Data are mean ± standard deviation. Significant *P* < 0.05

*LWR* lipid water ratio, *FF* fat fraction

### Logistic regression analysis and diagnostic performance of MRS, IVIM-DWI, and IVIM-DWI combined with MRS

As the parameter of FF was calculated according to the following equation: FF= LWR/(LWR +1), the ROC of MRS for differentiation between osteoporotic and osteolytic metastatic VCFs was drawn by FF. The sensitivity, specificity, and accuracy of MRS in differentiating osteoporotic from osteolytic metastatic VCFs were 87.50% (28/32), 57.89 (22/38), and 71.43% (50/70), respectively (Table 4).

**Table 4:** Diagnostic performances of the MRS, IVIM-DWI, and IVIM-DWI combined with MRS

	Sensitivity (%)	Specificity (%)	Accuracy (%)	AUC (95% CI)	*P*_*1*_	*P*_*2*_	*P*_*3*_
MRS	87.50 (28/32)	57.89 (22/38)	71.43 (50/70)	0.730 (0.589–0.812)	0.026	0.000	0.046
IVIM-DWI	78.13 (25/32)	89.47 (34/38)	84.28 (59/70)	0.875 (0.772–0.941)			
IVIM-DWI combined with MRS	90.63 (29/32)	97.37 (37/38)	94.29 (66/70)	0.964 (0.889–0.994)			

*P*_*1*_ the difference between MRS and IVIM_DWI, *P*_*2*_ the difference between MRS and IVIM_DWI combined with MRS*, P*_*3*_ the difference between IVIM_DWI and IVIM-DWI combined with MRS

On multivariate logistic regression analysis, *D*, D^*^ and *f* values were independent predictors for the diagnosis of osteoporotic and osteolytic metastatic VCFs with IVIM-DWI. The diagnostic equation was as follows:

$$ \mathrm{Logit}\left(\mathrm{P}1\right)=-3.068-736.188\ast \mathrm{D}+135.729\ast \mathrm{D}\ast \hbox{-} 8.449\ast \mathrm{f} $$

Using this regression equation to evaluate the 70 VBFs (*P*> 0.5 as metastatic, *P*≤ 0.5 as osteoporotic), the sensitivity, specificity, and accuracy of IVIM-DWI in differentiating osteoporotic from osteolytic metastatic VCFs were 78.13% (25/32), 89.47 (34/38), and 84.28 (59/70), respectively (Table 4).

After logistic regression analysis, FF, *D*, D^*^, and *f* values were identified as independent predictors of a diagnosis of osteoporotic and osteolytic metastatic VCFs with IVIM-DWI combined with MRS. The following diagnosis equation was used.

$$ \mathrm{Logit}\left(\mathrm{P}2\right)=-0.859-0.172\ast \mathrm{FF}-82.869\kern0.5em \ast \mathrm{D}+144.717\kern0.5em \ast \mathrm{D}\ast -9.847\ast \mathrm{f} $$

Using this regression diagnosis equation to evaluate the 70 VCFs (*P*> 0.5 as metastatic, *P*≤ 0.5 as osteoporotic), the diagnostic accuracy was 94.29 (66/70). The diagnostic sensitivity and specificity were 90.63% (29/32) and 97.37 (37/38), respectively (Table 4).

### Diagnostic performance of MRS, IVIM-DWI, and IVIM-DWI combined with MRS

**Fig. 4:**
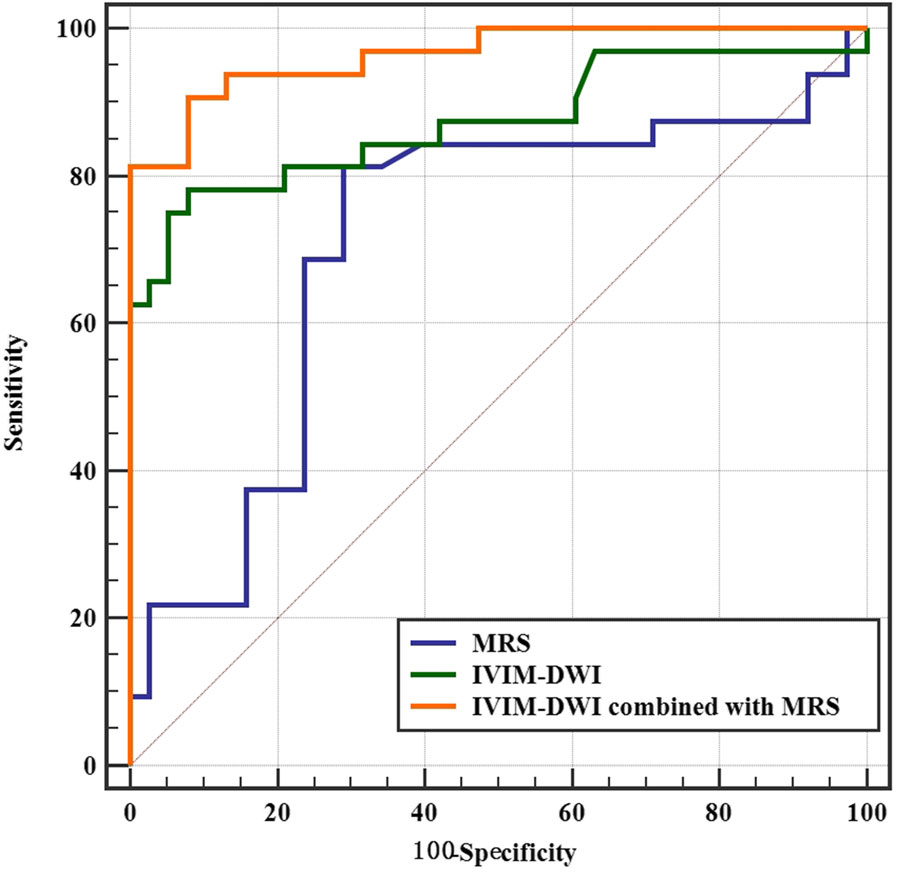
Receiver operating characteristic (ROC) curves revealed the diagnostic performances of MRS, IVIM-DWI, and IVIM-DWI combined with MRS in differentiating metastatic fractures from osteoporotic vertebral compression fractures

The AUC of MRS, IVIM-DWI, and MRS combined with IVIM-DWI were 0.73 (95% CI 0.589–0.812), 0.88 (95% CI 0.772–0.941), and 0.94 (95% CI 0.88–0.994), respectively (*P*<0.05) (Table 4). Furthermore, the comparison of the receiver operating characteristic (ROC) showed that IVIM-DWI combined with MRS has the best diagnostic performance followed by the IVIM-DWI and MRS (Fig. 4).

## Discussion

Identification of the nature of VCFs is a common clinical problem, especially among those elderly prone to osteoporotic compression fractures; thus, the precise diagnosis can make choices for the treatment and prognosis. Although some morphological features have been reported to help identify the nature of fractures, the morphological features of osteoporotic vertebral fractures include fracture fragments, residual bone marrow, hyperintensity on STIR-weighted images, and no enhancement on contrast MRI [19]. The morphological features of malignant vertebral fractures include cortical swelling of the posterior vertebral body, epidural space-occupying, vertebral pedicle destruction, diffuse hypointensity on T1WI, and enhancement on contrast MRI. However, there lacks specificity among the morphological features reported.

The MRS can detect the ratio of lipid-to-water accurately, revealing the myeloid lipid content at the cellular level [20]. The quantitative parameters *f*, *D*, and D* from IVIM-DWI are used to assess the diffusion and microvascular perfusion of water molecules in the tissue, which can more accurately reflect the physiological and pathological conditions of the tissue. It has been widely used in various malignant tumor diagnosis [21, 22], which makes the differential diagnosis of vertebral fracture possible. However, it is rarely reported as the diagnostic performance of IVIM-DWI combined with MRS in distinguishing osteoporotic fractures from metastatic VCFs.

In our study, the value of FF in the malignant group was significantly lower than that in the benign group, which is consistent with the previous results [23, 24]. As fat content increase with age, the fat cells will replace the lost trabecular meshwork especially in osteoporosis patients. Among patients with osteoporotic fracture, the proportion of free water increase and T2-weighted image (STIR) shows a high signal intensity, and some residual lipids are still present in the vertebral body; MRS shows a decrease in peak height and peak area of the signals of lipid and an increase in peak height and peak area of the signals of water. On the other hand, with the increasing of vertebral edema or severe acute compression fractures, the vertebral body lipid composition is completely replaced by water or hematoma; MRS shows lipid peak flat, while water peak high and sharp. In our study, 16 cases of osteoporotic fractures are diagnosed as metastatic fractures by MRS. In the metastatic fractures, malignant cells can proliferate rapidly, bone marrow lipocytes are replaced by tumor cells, a lot of trabecular bone is damaged, bone integrity is weakened, and pathological fractures are easy to occur. MRS also shows peak flat, while water peak high and sharp, which has a certain specificity. However, it is difficult to identify among some osteoporotic fractures. Only 4 cases of metastatic fractures are diagnosed as osteoporotic fractures in our study. The results show that the MRS in diagnosing vertebral fractures has high sensitivity, but the specificity and accuracy were low. The area under the curve suggested that MRS has lower diagnostic efficiency to osteoporotic and metastatic vertebral fractures.

Using a single exponential model, the ADC value of benign fractures is higher than that of malignant fractures, and the ADC threshold for the diagnosis of malignant vertebral fractures is 1.7 × 10^−3^ mm^2^/s [25]. The ADC values reflect the degree of diffusion of water molecules, but the diagnostic accuracy of ADC values may be decreased in rich perfusion malignant tumors. In our study, a double exponential model of multi-*b* value DWI was applied to find out that the *D* value in metastatic group was lower than that in osteoporotic group, which may be due to malignant tumor cell density and small extracellular space. The increased viscosity of the cell membrane reduces the permeability which may slow down and restrict the water molecule diffusion. The results of lower *f* value and higher D* value in osteoporotic group were supposed to be due to the rapid growth of tumor angiogenesis and microvascular perfusion of metastatic vertebral fractures. However, osteoporotic vertebral fractures have no neovascularization, and blood perfusion is small [26]. In our study, 4 cases of osteoporotic fractures are diagnosed as metastatic fractures and 7 cases of metastatic fractures as osteoporotic fractures. It may be due to that some of the primary tumor of metastatic vertebral fractures were small cell type with only a few newborn microvasculature, so there was some overlap among the *D* value of metastatic vertebral fractures and osteoporotic fractures. The sensitivity, specificity, and accuracy of IVIM in the diagnosis of vertebral fracture are 78.13%, 89.47%, and 84.28%, respectively, and the AUC is 0.88, indicating that IVIM has higher diagnostic performance for osteoporotic and metastatic vertebral fractures; however, its sensitivity was low.

The sensitivity, specificity, and accuracy of combining MRS and IVIM-DWI in the diagnosis of vertebral fracture were 90.63%, 97.37%, and 94.29%, respectively, and AUC was the highest (0.94), which suggested that the diagnostic performance was significantly improved. Among 38 osteoporotic fractures, 16 cases were diagnosed as metastatic by MRS, and 12 cases were diagnosed as osteoporotic with a combination of IVIM-DWI and MRS, suggesting that IVIM-DWI could differentiate the nature of vertebral fracture and provided valuable information according to the viewpoint of restricted diffusion of water molecules. Among 32 metastatic fractures, 4 cases were diagnosed as osteoporotic fracture by IVIM, and 3 cases were diagnosed as malignant by IVIM-DWI combined with MRS, because the primary tumors were prostate cancer and small cell lung cancers, and the changes in cell density of these two types of metastatic diseases were not obviously significant. It was easy to misdiagnose the nature of vertebral fracture which only based on multi-*b* value IVIM diffusion-weighted images. IVIM-DWI combined with MRS could increase the diagnosis confidence.

There were several limitations to our study. Firstly, metastatic vertebral fractures originate from different types of primary tumors and are not grouped according to the primary malignancy, which may lead to different perfusion patterns and signal differences. Secondly, there were different diffusions between osteogenic and osteolytic metastasis; therefore, the study excluded osteogenic metastases, and the group of metastatic vertebral fractures are mostly osteolytic metastasis; osteogenic metastasis could be incorporated into the study as one of the research directions in the future. Thirdly, the majority of diagnoses were based on clinical and radiographic evidence that biopsy confirms that the nature of the fractures was not routinely performed. Finally, other benign and malignant lesions, such as spondylitis (suppurative or tuberculous), primary vertebrae body tumors, and multiple myeloma were not included in this study.

## Conclusion

Overall, IVIM-DWI combined with MRS can improve the differential diagnostic performance of osteoporotic and osteolytic metastatic vertebral fractures, which is of great significance to guide the clinical development of the correct treatment plan.

## Data Availability

The datasets used and/or analyzed during the current study are available from the corresponding author on reasonable request.

## Abbreviations

IVIM-DWI: Intravoxel incoherent motion diffusion-weighted MR imaging
MRS: Magnetic resonance spectroscopy
VCFs: Vertebral compression fractures
*D*: Diffusion coefficient
*D**: Pseudo diffusion
*f*: Perfusion fraction
LWR: Lipid water ratio
FF: Fat fraction
ROC: Receiver operating characteristic
